# Converging physiological roles of the anthrax toxin receptors

**DOI:** 10.12688/f1000research.19423.1

**Published:** 2019-08-12

**Authors:** Oksana A. Sergeeva, F. Gisou van der Goot

**Affiliations:** 1 Global Health Institute, School of Life Sciences, EPFL, Lausanne, Switzerland

**Keywords:** anthrax toxin receptors, CMG2, TEM8, HFS, ISH, JHF, GAPO

## Abstract

The anthrax toxin receptors—capillary morphogenesis gene 2 (CMG2) and tumor endothelial marker 8 (TEM8)—were identified almost 20 years ago, although few studies have moved beyond their roles as receptors for the anthrax toxins to address their physiological functions. In the last few years, insight into their endogenous roles has come from two rare diseases: hyaline fibromatosis syndrome, caused by mutations in CMG2, and growth retardation, alopecia, pseudo-anodontia, and optic atrophy (GAPO) syndrome, caused by loss-of-function mutations in TEM8. Although CMG2 and TEM8 are highly homologous at the protein level, the difference in disease symptoms points to variations in the physiological roles of the two anthrax receptors. Here, we focus on the similarities between these receptors in their ability to regulate extracellular matrix homeostasis, angiogenesis, cell migration, and skin elasticity. In this way, we shed light on how mutations in these two related proteins cause such seemingly different diseases and we highlight the existing knowledge gaps that could form the focus of future studies.

## Introduction

In the early 2000s, two newly discovered proteins—capillary morphogenesis gene 2 (CMG2) and tumor endothelial marker 8 (TEM8)—were demonstrated to be anthrax toxin receptors (ANTXRs) ^1, 2^. Since then, much research has focused on their toxin-related pathogenic roles (most recently reviewed by Friebe *et al*. ^3^ and Sun and Jacquez ^4^). However, both CMG2, encoded by the *ANTXR2* gene, and TEM8, encoded by the *ANTXR1* gene, also possess physiological roles in vertebrates, the study of which has not received nearly the same level of attention. What has helped to drive research into their endogenous roles is that both receptors are associated with recessive autosomal diseases: mutations in CMG2 lead to hyaline fibromatosis syndrome (HFS), whereas mutations in TEM8 result in growth retardation, alopecia, pseudo-anodontia, and optic atrophy (GAPO) syndrome ^5– 7^. The literature contains reports of some 350 HFS patients of whom 112 have been genotyped, possessing 56 different mutations. In parallel, 70 GAPO patients have been reported, 21 of which have genotype information, comprising 14 different mutations. Even though these diseases have grossly different symptoms, CMG2 and TEM8 share 62% sequence similarity, reasonably allowing for some overlapping structure/function relationship and ensuring that a better understanding of the function of one receptor can prompt progress on the other. Therefore, this review aims to synthesize the latest information on these ANTXRs with earlier established observations to better understand their physiological functions and provide open pathways for future research.

## Anthrax toxin receptor structure and epistructure

**Figure 1.**
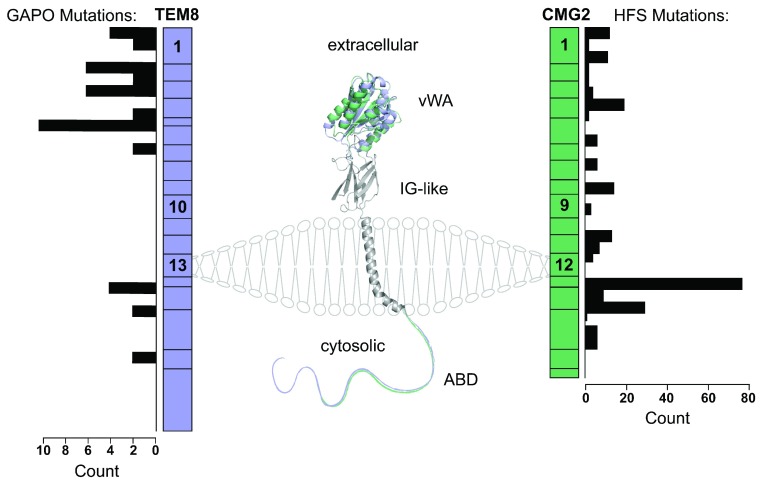
TEM8 and CMG2 are similar in gene and protein structure: mutations in the former lead to GAPO and those in the latter lead to HFS. Tumor endothelial marker 8 (TEM8) (blue) and capillary morphogenesis gene 2 (CMG2) (green) have similar exon schemes and protein structures. Crystal structures of the von Willebrand factor type A (vWA) domain of TEM8 (Protein Data Bank [PDB]: 3N2N ^8^) and CMG2 (PDB: 1TZN ^9^) are shown aligned. The immunoglobulin (Ig)-like and transmembrane domains have been modelled on CMG2 and are shown in gray ^10^. The cytosolic tails, longer for TEM8 than for CMG2, are intrinsically disordered with a conserved juxtamembranous actin-binding domain (ABD). The number of reported occurrences of mutations in growth retardation, alopecia, pseudo-anodontia, and optic atrophy (GAPO) is depicted next to TEM8, and the corresponding number in hyaline fibromatosis syndrome (HFS) is shown next to CMG2. The number of HFS patients with mutations in CMG2 is almost an order of magnitude higher than those of GAPO/TEM8.

Both CMG2 and TEM8 are type I transmembrane proteins consisting of an extracellular von Willebrand factor type A (vWA) that is also found in integrins and participates in receptor–ligand interactions, an extracellular immunoglobulin-like (Ig-like) domain, and a long cytoplasmic tail that is predicted to be largely unstructured and contains an actin–cytoskeleton interacting domain ( Figure 1). The structure of the extracellular domains was analyzed by low-resolution cryo-electron microscopy, onto which the x-ray structure of the vWA domain ^8, 9^ and a model of the Ig-like domain ^10, 11^ were successfully docked. CMG2 and TEM8 have long cytosolic tails of 148 and 222 residues, respectively, which are predicted to be intrinsically unstructured like the cytoplasmic domains of many other signaling receptors ^12^. These tails could allow sequential interaction with diverse partner molecules ^13^ and were also found to be the site of various post-translational modifications, such as S-palmitoylation ^14^, ubiquitination ^14^, and tyrosine-phosphorylation ^15^, all of which were found to be necessary for toxin-induced endocytosis ^3^. The CMG2 and TEM8 tails are completely identical in certain segments but have no homology in others ^16^, pointing toward similarities and differences, respectively, in interacting partners. The most highly conserved portion between the two receptors, which is juxtamembrane, has homology with the actin-regulating Wiskott–Aldrich syndrome protein ^17^. Relatedly, TEM8 was also consistently shown to interact with actin ^18, 19^, although this interaction may be indirect and require adaptor proteins, such as those observed for integrins ^20^.

## Receptors for anthrax toxin

**Figure 2.**
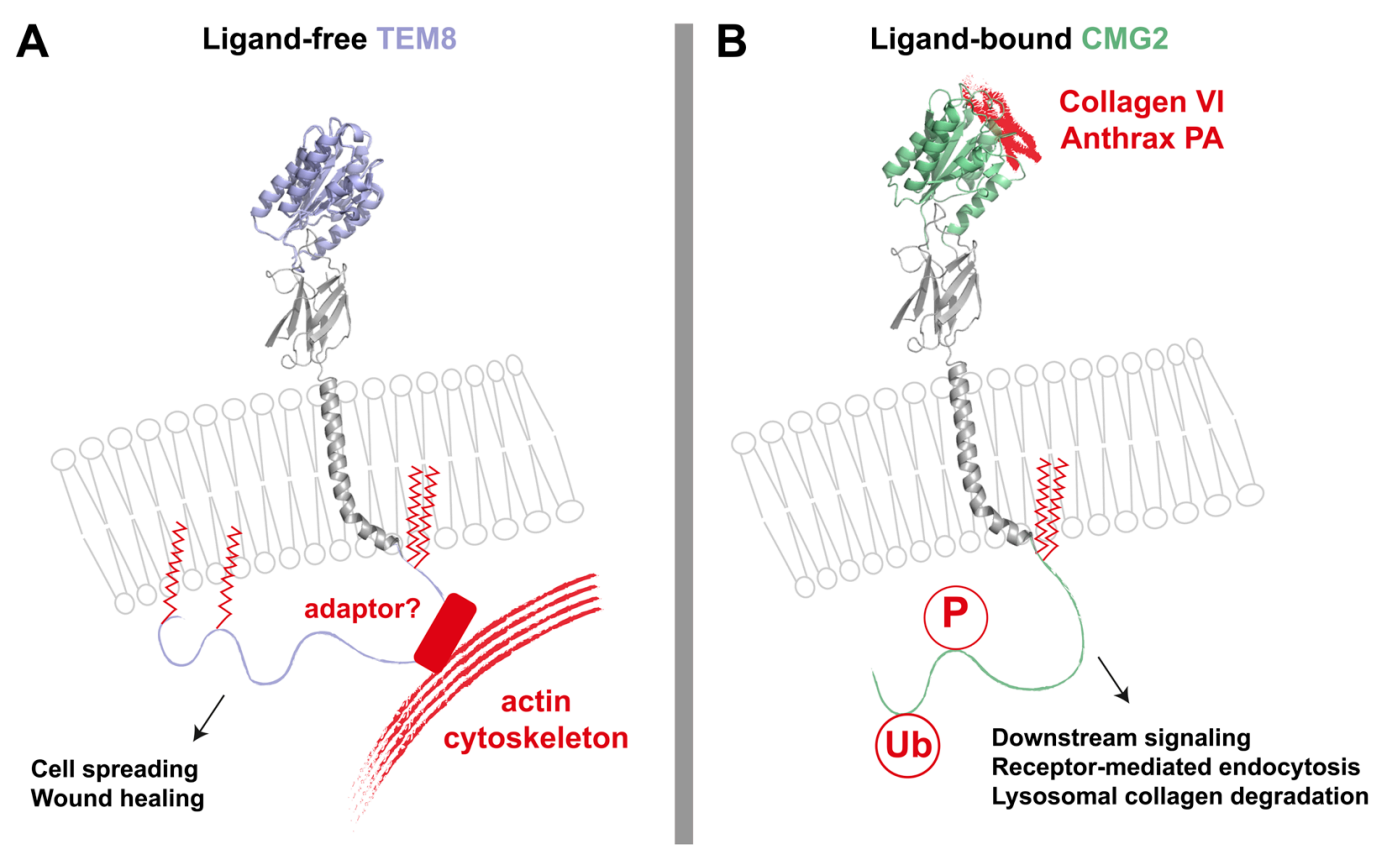
Depictions of ligand-free TEM8 and ligand-bound CMG2. ( **A**) Ligand-free tumor endothelial marker 8 (TEM8) is palmitoylated and bound directly or indirectly to the actin cytoskeleton. Through this association, it plays a role in cell spreading and migration as well as wound healing. Red zigzag lines represent S-palmitoylation modifications of TEM8, which increase resident time of either receptor at the cell surface. ( **B**) When bound to collagen VI (ColVI) or anthrax toxin protective antigen (PA), capillary morphogenesis gene 2 (CMG2) becomes phosphorylated (“P”) and ubiquitinated (“Ub”). This allows CMG2 to signal downstream within the cell, endocytose the receptor–ligand complex, and degrade ColVI in the lysosomes.

The function of CMG2 and TEM8 as ANTXRs has been extensively studied. The surface residence time of ANTXRs is regulated by S-palmitoylation at three or four sites in their cytoplasmic tails, as shown for TEM8 ^14^, and under “resting” conditions, TEM8 is associated with the actin cytoskeleton ^15, 21^ ( Figure 2A). As opposed to how integrins concurrently interact with their ligand and the cytoskeleton, upon binding of a ligand such as anthrax toxin protective antigen (PA), the interaction of TEM8 with the actin cytoskeleton is released. Upon ligand binding, PA oligomerizes into a heptameric or octameric complex, leading to clustering of the receptors ^2, 22, 23^. This, in turn, triggers *src* family kinase-mediated phosphorylation of cytoplasmic tyrosines in the ANTXR tails ^15, 24^ and subsequent recruitment of the adaptor protein β-arrestin, allowing an E3 ligase (Cbl for TEM8) to bind and facilitate ubiquitination ^14, 24^. Ubiquitinated ligand-bound ANTXRs are taken up by an adaptor protein 1 (AP-1)-dependent and clathrin-mediated endocytic route ^24^. Upon arrival in sorting endosomes, in the presence of a multivalent ligand, ANTXRs are sorted into nascent intraluminal vesicles. The exact mechanism and molecular requirements for this sorting have not been investigated. Although a bird’s-eye view of toxin-induced endocytosis is available, little is known about the physiological endocytic trafficking of ANTXRs, particularly whether they undergo endocytosis and recycling for re-utilization.

## HFS-causing CMG2 mutations

HFS is now the unifying term for two diseases previously described in the literature: infantile systemic hyalinosis (ISH), which was named in 1986 ^25^, and juvenile hyaline fibromatosis (JHF), first described in 1873 by Murray ^26^ and named in 1976 ^27^. Initially, these terms were thought to describe two different diseases with overlapping symptoms, including subcutaneous nodules, gingival hypertrophy, painful joint contractures, and persistent infections. ISH is, however, more severe with death occurring before the age of two because of protein-losing enteropathy. In the early 2000s, patients with both JHF and ISH were found to have mutations in CMG2 ^5, 6, 28^, pointing to the disease causality. This allowed the classification of a unified syndrome with a symptom grading system, placing the previously named ISH and JHF on two ends of a continuum of disease severity ^29, 30^. We strongly encourage the community to adopt a single nomenclature—HFS instead of ISH or JHF—to ensure clarity in the literature.

HFS-causing mutations in CMG2 fall into four classes: (a) missense mutations in the vWA domain that specifically affect ligand binding; (b) missense mutations in exons 1 to 11 that affect folding/stability of the ectodomain, leading to protein degradation; (c) frameshift mutations that lead to premature stop codons, nonsense mutations, and those that affect splicing, leading to rapidly degraded mRNA; and (d) missense mutations in the cytosolic tail that do not affect protein abundance or localization but likely affect some aspect of CMG2 function ^16^. Casas-Alba *et al*. recently published an exhaustive review on phenotype–genotype correlations of patients with HFS ^31^. The authors used the aforementioned grading system ^30^ to support the general notion that missense mutations in exons 1 to 12 and nonsense and frameshift mutations lead to more severe forms of the disease than missense mutations in exons 13 to 17 ^31^. The gravest mutations largely affect protein abundance due to mRNA or protein degradation, whereas the milder mutations do not affect protein expression levels ^16^. One exception is the missense mutations that prevent ligand binding, which still produce normal amounts of protein but are even more severe than mutations that lead to near-complete protein loss. This observation raises interesting possibilities. Either ligand-binding-deficient CMG2 not only loses its initial function but may gain a new pathogenic one or unliganded CMG2 has important signaling functions that need to be switched off by ligand binding. Upon mutation of the ligand binding site, sustained signaling could be detrimental.

## GAPO-causing TEM8 mutations

The symptoms of GAPO were first described by Andersen and Pindborg in 1947 ^32^ but the term was not coined until 1984 ^33^. The name of the disease itself, GAPO, describes its most characteristic symptoms: growth retardation, alopecia, pseudo-anodontia, and optic atrophy; however, since optic atrophy is seen in only a small fraction of patients, there is an appeal to rename the O to “ocular manifestations”, as many patients do have other eye defects, such as glaucoma ^34^. Although only a few cases of GAPO lead to death before adulthood ^34– 36^, it is still a severe disease that often gives patients a geriatric appearance. The link between GAPO manifestation and mutations in TEM8 was found only in 2013 ^7^, 10 years after the connection between CMG2 and HFS was discovered.

Recently, Abdel-Hamid *et al*. reported on seven new GAPO patients and their associated mutations ^37^, almost doubling the known mutations. It is still premature to identify any mutational hot spots in TEM8 like we observe in the juxtamembranous exon 13 of CMG2 for HFS ( Figure 1). However, the mutant categories for GAPO will likely be similar to those of HFS, with missense mutations that map to the TEM8 cytosolic tail leading to milder phenotypes, as suggested by the description of a homozygous patient presenting with pseudo-anodontia and no other reported symptoms ^38^.

## Converging physiological functions of anthrax toxin receptors

### Extracellular matrix homeostasis and remodeling

What is clearest about the physiological roles of CMG2 and TEM8 is that they both interact with the extracellular matrix (ECM), although the specific ECM–protein interaction partners have been a point of contention in the literature. Early *in vitro* studies reported that CMG2 can bind collagen (Col) IV and laminin ^17^. That experiment relied on testing five ECM proteins (those two and osteopontin, fibronectin, and albumin) ^17^, overlooking other potential ECM ligands. Recent and more comprehensive *in vitro* studies have instead indicated that CMG2 has a higher affinity for ColVI than ColIV or laminin ^39^. TEM8 was also found to bind the cleaved C5 domain of ColVI(α3) ^40^. In mice, the lack of CMG2 or TEM8 can lead to an accumulation of ECM in various tissues, and there is some controversy as to which components accumulate. In mice lacking CMG2, an accumulation of only ColVI ^39^ or both ColI and ColVI ^41^ was observed, whereas in mice lacking TEM8, an increase in both ColI and ColVI was detected ^42, 43^.

Significant insight into the physiological function of CMG2 and its involvement in HFS has come from the recent work of Bürgi *et al*., who analyzed HFS patient nodules to reveal that they are predominantly made up of ColVI ^39^. The authors also showed that CMG2 is a cellular receptor for this non-fibrillar collagen and that it can mediate the degradation of ColVI in lysosomes ( Figure 2B) ^39^. Strikingly, they observed that whereas female mice lacking CMG2 have hypertrophic uteri and parturition defects (seen previously ^41, 44^), mice deficient for both CMG2 and ColVI were able to deliver pups normally ^39^. Furthermore, this study did not implicate matrix metalloprotease inhibition as a mechanism for extracellular ColVI accumulation, as was proposed in earlier studies ^41, 43, 45^. Thus, it appears to be the inability of CMG2 to control the abundance of ColVI in the extracellular space that leads to the formation of nodules in patients with HFS. Explaining other HFS symptoms, such as life-threatening protein-losing enteropathy in infants, will require further investigation.

The fact that CMG2 is a bona fide ColVI receptor is new, although the literature had previously hypothesized that the ANTXRs regulate collagen clearance, and CMG2 itself was already thought to be the cellular receptor responsible for collagen internalization and degradation ^44^. Meanwhile, TEM8 was postulated to be involved in collagen degradation through an endocytosis-mediated pathway ^46^, and TEM8 recycling was posited to lead to ColI and ColVI clearance ^47^. In one of the earlier reports of GAPO, Wajntal *et al*. theorized that the disease symptoms were due to ECM accumulation and hypothesized that GAPO was the result of an autosomal recessive defect in a gene responsible for ECM component breakdown ^48^. However, TEM8 is unable to compensate for ColVI degradation in human fibroblasts upon silencing of CMG2 or in cells of patients with HFS, suggesting that even though TEM8 might have the ability to bind ColVI, it does not escort it to lysosomes, at least not in fibroblasts, as CMG2 does ^39^. Since TEM8 was found to bind the C5 domain of ColVI, which is processed during maturation, TEM8 might have a different role in ColVI homeostasis.

### Angiogenesis and cancer

Both CMG2 and TEM8 were originally discovered because of their connection to angiogenesis. In 2000, TEM8 was found to be the eighth most upregulated marker in the tumor endothelium ^49^, and in 2001, the gene encoding for CMG2 was uncovered as the second most upregulated gene in *in vitro* capillary morphogenesis in three-dimensional collagen matrices ^17^. Consistent with these findings, anthrax PA was observed to prevent angiogenesis in a moderate yet significant manner ^50^. Additionally, a heterozygous mutation in the transmembrane domain of TEM8 is associated with infantile hemangiomas, benign tumors arising from disorganized angiogenesis ^51^.

Since angiogenesis is a major hallmark of cancer, researchers have investigated the roles of CMG2 and TEM8 in cancer since their initial discovery. For TEM8, the literature is consistent in that its overexpression results in more aggressive cancer, especially for osteosarcomas ^52^, gallbladder carcinomas ^53^, melanomas ^42^, and lung ^54^, breast ^55, 56^, and colorectal ^57^ cancers. However, for CMG2, there are mixed reports of the effect of its overexpression on cancer progression; some show that lower CMG2 correlated with a more aggressive soft-tissue sarcoma ^58^ and breast cancer ^59^, and others reveal that higher CMG2 resulted in a worse survival rate in patients with gastric cancer ^60^ and glioblastoma ^61^. Although these studies link these proteins to angiogenesis, mechanistic explanations of the cancer-related roles of ANTXRs are still lacking. Research has also focused on targeting CMG2 or TEM8 for cancer therapy ^62^, especially using anti-TEM8 antibodies ^63, 64^, CMG2 vWA domain fragments ^65^, or anthrax toxin itself ^66^ for slowing cancer progression. Recently, Szot *et al*. demonstrated the potential of delivering antibody drug conjugates targeting TEM8 to tumor-associated stromal cells, thereby unexpectedly but effectively killing nearby cancer cells ^67^. Meanwhile, Byrd *et al*. successfully used TEM8 chimeric antigen receptor T cells to target and regress xenograft tumors derived from patients with triple-negative breast cancer ^68^.

### Actin cytoskeleton and cell migration

As mentioned earlier, both ANTXRs contain a putative actin-interacting peptide sequence in their cytoplasmic tails (juxtamembrane residues 355 to 420), and it has been shown that ligand-free TEM8 binds to the actin cytoskeleton ^24^ ( Figure 2A). Furthermore, it was proposed that the actin cytoskeleton can regulate the affinity of TEM8 for its extracellular ligands ^21, 69^. More specifically, Go *et al*. showed that mutating tyrosine 383 to cysteine in TEM8 (mimicking an HFS mutation, Y381C) decreased actin–cytoskeleton interactions but increased anthrax-toxin binding ^69^. This type of inside-out signaling is reminiscent of integrins. The fact that integrins are involved in cell adhesion, spreading, and migration has led to studies asking whether TEM8 could be involved in similar cellular processes. TEM8 was reported to affect cell adhesion and spreading by coupling the ECM with the actin cytoskeleton ^19, 47^. However, while integrins simultaneously bind extracellular ligands and the intracellular actin cytoskeleton, thus generating force, the mutually exclusive interaction of TEM8 with ligands and actin indicates that the contribution to spreading must occur via different mechanisms, which remain to be elucidated.

One process that combines angiogenesis, cell migration, and ECM remodeling is wound healing. In 2016, Wang *et al*. demonstrated that TEM8 expression is increased in acute or chronic wounds as compared with normal skin ^70^. When they depleted TEM8 in keratinocyte cells, they saw decreased migration and proliferation ^70^. However, the exact molecular role of TEM8 in regulating wound healing is still unknown.

Surprisingly, CMG2 has not yet been characterized to interact with the actin cytoskeleton. Instead, zebrafish Antxr2a, a CMG2 ortholog, was shown to be involved in positioning the mitotic spindle in a process that involves the small GTPase RhoA and its downstream effector mDia ^71^. Consistent with a putative role of CMG2 in actin cytoskeleton rearrangements, CMG2 knockdown led to decreased migration in human uterine smooth muscle cells while its overexpression led to increased migration ^45^.

### Skin physiology and organ fibrosis

Both HFS and GAPO patients have issues with the skin: skin thickening in HFS and alopecia in GAPO. Although few histopathological analyses have been reported for patients with GAPO ^37^, the dermis of one patient with GAPO was shown to have abundant hyaline material with increased collagenous fibers ^48^, suggesting that, like HFS (the earliest histopathological analyses ^72^ to the latest ^73^), GAPO is a disease of the connective tissue. This increased ECM could interfere in the development and cycling of the hair follicles ^74^ but this is still unproven.

An analysis of TEM8 knockout (KO) mice was consistent with patient molecular observations in that increased ECM is seen in organs that consequently develop fibrosis ^42, 43^. Whereas earlier reports did not observe gross changes in the TEM8 KO mice ^42^, more recent studies on a different KO mouse line indicate that mice rather accurately mimic the GAPO phenotype ^43^. Hu *et al*. recently showed that fibroblasts isolated from older mice deficient in TEM8 have an increased expression of ColI, fibronectin, and the connective tissue growth factor (CTGF) ^75^. They argue for a cell-autonomous mechanism wherein TEM8 targets CTGF to regulate ECM production, meaning that TEM8-lacking cells have higher CTGF, thus a higher ECM production that leads to fibrosis ^75^.

It is important to note that nodules develop months or years after birth in patients with HFS and that hair loss starts around age two for patients with GAPO ^16, 37^. Similarly, ECM accumulation was predominantly seen in older patients with GAPO ^7^. In mice lacking CMG2, the uterine fibrosis phenotype was seen only in sexually mature mice ^41^; similarly, progressive fibrosis was observed for mice deficient for TEM8 ^43^. This could mean either that the organism can temporarily buffer disease or that the accumulation of ECM reaches a threshold that causes these manifestations. One obvious implication is that CMG2 might partly compensate for TEM8 function and vice versa before the cell is overwhelmed and the disease symptoms arise. Interestingly, double-KO mice are viable but produce no pups when mated ^76^, suggesting that the receptors have non-redundant roles in fertility, embryonic development, or parturition.

## Conclusions and Outlook

In recent years, significant progress has been made in understanding the functions of CMG2 and TEM8 in vertebrates. Specifically, the fact that these two receptors act as causative genes for strikingly different diseases was perplexing, as their main domains are conserved so they likely have similar physiological roles in the cell. The similarity in underlying molecular defects—both receptors contribute to ECM homeostasis, angiogenesis, cell migration, and skin pathology—has helped to explain this. However, the devil appears to be in the details that remain elusive: which collagen(s) do they bind, how do they (directly) interact with actin, can they endocytose ECM components for lysosomal breakdown without depleting the extracellular environment of their ligand, which signaling cascades do they trigger, and in which cells do they primarily function? Furthermore, the role that these ANTXRs play at a molecular level in angiogenesis, cell migration, and wound healing remains to be elucidated. We hope that this review has provided sufficient evidence of the analogous nature of the ANTXRs to guide future research into these two receptors.

## Abbreviations

ANTXR, anthrax toxin receptor; CMG2, capillary morphogenesis gene 2; Col, collagen; CTGF, connective tissue growth factor; ECM, extracellular matrix; GAPO, growth retardation, alopecia, pseudo-anodontia, and optic atrophy; HFS, hyaline fibromatosis syndrome; Ig, immunoglobulin; ISH, infantile systemic hyalinosis; JHF, juvenile hyaline fibromatosis; KO, knockout; PA, protective antigen; TEM8, tumor endothelial marker 8; vWA, von Willebrand factor type A

## References

[1] Scobie HM Rainey GJ Bradley KA: Human capillary morphogenesis protein 2 functions as an anthrax toxin receptor. *Proc Natl Acad Sci U S A.* 2003;100(9):5170–4. 10.1073/pnas.0431098100 12700348 PMC154317

[2] Bradley KA Mogridge J Mourez M: Identification of the cellular receptor for anthrax toxin. *Nature.* 2001;414(6860):225–9. 10.1038/n35101999 11700562

[3] Friebe S van der Goot F Bürgi J: The Ins and Outs of Anthrax Toxin. *Toxins (Basel).* 2016;8(3): pii: E69. 10.3390/toxins8030069 26978402 PMC4810214

[4] Sun J Jacquez P: Roles of Anthrax Toxin Receptor 2 in Anthrax Toxin Membrane Insertion and Pore Formation. *Toxins (Basel).* 2016;8(2):34. 10.3390/toxins8020034 26805886 PMC4773787

[5] Dowling O Difeo A Ramirez MC: Mutations in capillary morphogenesis gene-2 result in the allelic disorders juvenile hyaline fibromatosis and infantile systemic hyalinosis. *Am J Hum Genet.* 2003;73(4):957–66. 10.1086/378781 12973667 PMC1180616

[6] Hanks S Adams S Douglas J: Mutations in the gene encoding capillary morphogenesis protein 2 cause juvenile hyaline fibromatosis and infantile systemic hyalinosis. *Am J Hum Genet.* 2003;73(4):791–800. 10.1086/378418 14508707 PMC1180602

[7] Stránecký V Hoischen A Hartmannová H: Mutations in *ANTXR1* cause GAPO syndrome. *Am J Hum Genet.* 2013;92(5):792–9. 10.1016/j.ajhg.2013.03.023 23602711 PMC3644626

[8] Fu S Tong X Cai C: The structure of tumor endothelial marker 8 (TEM8) extracellular domain and implications for its receptor function for recognizing anthrax toxin. *PLoS One.* 2010;5(6):e11203. 10.1371/journal.pone.0011203 20585457 PMC2887854

[9] Lacy DB Wigelsworth DJ Melnyk RA: Structure of heptameric protective antigen bound to an anthrax toxin receptor: a role for receptor in pH-dependent pore formation. *Proc Natl Acad Sci U S A.* 2004;101(36):13147–51. 10.1073/pnas.0405405101 15326297 PMC516539

[10] Deuquet J Lausch E Guex N: Hyaline fibromatosis syndrome inducing mutations in the ectodomain of anthrax toxin receptor 2 can be rescued by proteasome inhibitors. *EMBO Mol Med.* 2011;3(4):208–21. 10.1002/emmm.201100124 21328543 PMC3377065

[11] Jacquez P Avila G Boone K: The Disulfide Bond Cys255-Cys279 in the Immunoglobulin-Like Domain of Anthrax Toxin Receptor 2 Is Required for Membrane Insertion of Anthrax Protective Antigen Pore. *PLoS One.* 2015;10(6):e0130832. 10.1371/journal.pone.0130832 26107617 PMC4479931

[12] Bürgi J Xue B Uversky VN: Intrinsic Disorder in Transmembrane Proteins: Roles in Signaling and Topology Prediction. *PLoS One.* 2016;11(7):e0158594. 10.1371/journal.pone.0158594 27391701 PMC4938508

[13] Kjaergaard M Kragelund BB: Functions of intrinsic disorder in transmembrane proteins. *Cell Mol Life Sci.* 2017;74(17):3205–24. 10.1007/s00018-017-2562-5 28601983 PMC11107515

[14] Abrami L Leppla SH van der Goot FG: Receptor palmitoylation and ubiquitination regulate anthrax toxin endocytosis. *J Cell Biol.* 2006;172(2):309–20. 10.1083/jcb.200507067 16401723 PMC2063559

[15] Abrami L Kunz B van der Goot FG: Anthrax toxin triggers the activation of src-like kinases to mediate its own uptake. *Proc Natl Acad Sci U S A.* 2010;107(4):1420–4. 10.1073/pnas.0910782107 20080640 PMC2824395

[16] Deuquet J Lausch E Superti-Furga A: The dark sides of capillary morphogenesis gene 2. *EMBO J.* 2012;31(1):3–13. 10.1038/emboj.2011.442 22215446 PMC3252584

[17] Bell SE Mavila A Salazar R: Differential gene expression during capillary morphogenesis in 3D collagen matrices: regulated expression of genes involved in basement membrane matrix assembly, cell cycle progression, cellular differentiation and G-protein signaling. *J Cell Sci.* 2001;114(Pt 15):2755–73. 11683410 10.1242/jcs.114.15.2755

[18] Garlick KM Mogridge J: Direct interaction between anthrax toxin receptor 1 and the actin cytoskeleton. *Biochemistry.* 2009;48(44):10577–81. 10.1021/bi9015296 19817382 PMC2847348

[19] Werner E Kowalczyk AP Faundez V: Anthrax toxin receptor 1/tumor endothelium marker 8 mediates cell spreading by coupling extracellular ligands to the actin cytoskeleton. *J Biol Chem.* 2006;281(32):23227–36. 10.1074/jbc.M603676200 16762926

[20] Jeong SY Martchenko M Cohen SN: Calpain-dependent cytoskeletal rearrangement exploited for anthrax toxin endocytosis. *Proc Natl Acad Sci U S A.* 2013;110(42):E4007–E4015. 10.1073/pnas.1316852110 24085852 PMC3801034

[21] Garlick KM Batty S Mogridge J: Binding of filamentous actin to anthrax toxin receptor 1 decreases its association with protective antigen. *Biochemistry.* 2012;51(6):1249–56. 10.1021/bi2016469 22303962 PMC3286128

[22] Milne JC Furlong D Hanna PC: Anthrax protective antigen forms oligomers during intoxication of mammalian cells. *J Biol Chem.* 1994;269(32):20607–12. 8051159

[23] Molloy SS Bresnahan PA Leppla SH: Human furin is a calcium-dependent serine endoprotease that recognizes the sequence Arg-X-X-Arg and efficiently cleaves anthrax toxin protective antigen. *J Biol Chem.* 1992;267(23):16396–402. 1644824

[24] Abrami L Bischofberger M Kunz B: Endocytosis of the anthrax toxin is mediated by clathrin, actin and unconventional adaptors. *PLoS Pathog.* 2010;6(3):e1000792. 10.1371/journal.ppat.1000792 20221438 PMC2832758

[25] Landing BH Nadorra R: Infantile systemic hyalinosis: report of four cases of a disease, fatal in infancy, apparently different from juvenile systemic hyalinosis. *Pediatr Pathol.* 1986;6(1):55–79. 10.3109/15513818609025925 2434938

[26] Murray J: On three peculiar cases of Molluscum Fibrosum in Children in which one or more of the following conditions were observed: hypertrophy of the gums, enlargement of the ends of the fingers and toes, numerous connecive-tissue tumours on the scalp, &c. *Med Chir Trans.* 1873;56:235–254.1. 20896407 PMC1988914

[27] Kitano Y Horiki M Aoki T: Two cases of juvenile hyalin fibromatosis. Some histological, electron microscopic, and tissue culture observations. *Arch Dermatol.* 1972;106(6):877–83. 4118157

[28] Rahman N Dunstan M Teare MD: The gene for juvenile hyaline fibromatosis maps to chromosome 4q21. *Am J Hum Genet.* 2002;71(4):975–80. 10.1086/342776 12214284 PMC378553

[29] Nofal A Sanad M Assaf M: Juvenile hyaline fibromatosis and infantile systemic hyalinosis: a unifying term and a proposed grading system. *J Am Acad Dermatol.* 2009;61(4):695–700. 10.1016/j.jaad.2009.01.039 19344977

[30] Denadai R Raposo-Amaral CE Bertola D: Identification of 2 novel *ANTXR2* mutations in patients with hyaline fibromatosis syndrome and proposal of a modified grading system. *Am J Med Genet A.* 2012;158A(4):732–42. 10.1002/ajmg.a.35228 22383261 PMC4264531

[31] Casas-Alba D Martínez-Monseny A Pino-Ramírez RM: Hyaline fibromatosis syndrome: Clinical update and phenotype-genotype correlations. *Hum Mutat.* 2018;39(12):1752–63. 10.1002/humu.23638 30176098

[32] Andersen TH Pindborg JJ: A case of pseudoanodontia in connection with cranial deformity, nanosomia, and extodermal dysplasia. *Odontol Tidskr.* 1947;55:484–493.

[33] Tipton RE Gorlin RJ: Growth retardation, alopecia, pseudo-anodontia, and optic atrophy--the GAPO syndrome: report of a patient and review of the literature. *Am J Med Genet.* 1984;19(2):209–16. 10.1002/ajmg.1320190202 6507471

[34] Meguid NA Afifi HH Ramzy MI: GAPO syndrome: first Egyptian case with ultrastructural changes in the gingiva. *Clin Genet.* 1997;52(2):110–5. 10.1111/j.1399-0004.1997.tb02527.x 9298746

[35] Baxova A Kozlowski K Obersztyn E: GAPO syndrome (Radiographic clues to early diagnosis). *Radiol Med.* 1997;93(3):289–91. 9180938

[36] Demirgüneş EF Ersoy-Evans S Karaduman A: GAPO syndrome with the novel features of pulmonary hypertension, ankyloglossia, and prognathism. *Am J Med Genet A.* 2009;149A(4):802–5. 10.1002/ajmg.a.32686 19291762

[37] Abdel-Hamid MS Ismail S Zaki MS: GAPO syndrome in seven new patients: Identification of five novel *ANTXR1* mutations including the first large intragenic deletion. *Am J Med Genet A.* 2019;179(2):237–42. 10.1002/ajmg.a.61021 30575274

[38] Dinckan N Du R Akdemir ZC: A biallelic *ANTXR1* variant expands the anthrax toxin receptor associated phenotype to tooth agenesis. *Am J Med Genet A.* 2018;176(4):1015–22. 10.1002/ajmg.a.38625 29436111 PMC5933053

[39] Bürgi J Kunz B Abrami L: CMG2/ANTXR2 regulates extracellular collagen VI which accumulates in hyaline fibromatosis syndrome. *Nat Commun.* 2017;8:15861. 10.1038/ncomms15861 28604699 PMC5472780

[40] Nanda A Carson-Walter EB Seaman S: TEM8 interacts with the cleaved C5 domain of collagen alpha 3(VI). *Cancer Res.* 2004;64(3):817–20. 10.1158/0008-5472.CAN-03-2408 14871805

[41] Reeves CV Wang X Charles-Horvath PC: Anthrax toxin receptor 2 functions in ECM homeostasis of the murine reproductive tract and promotes MMP activity. *PLoS One.* 2012;7(4):e34862. 10.1371/journal.pone.0034862 22529944 PMC3328497

[42] Cullen M Seaman S Chaudhary A: Host-Derived Tumor Endothelial Marker 8 Promotes the Growth of Melanoma. *Cancer Res.* 2009;69(15):6021–6. 10.1158/0008-5472.CAN-09-1086 19622764 PMC2721800

[43] Besschetnova TY Ichimura T Katebi N: Regulatory mechanisms of anthrax toxin receptor 1-dependent vascular and connective tissue homeostasis. *Matrix Biol.* 2015;42:56–73. 10.1016/j.matbio.2014.12.002 25572963 PMC4409530

[44] Peters DE Zhang Y Molinolo AA: Capillary morphogenesis protein-2 is required for mouse parturition by maintaining uterine collagen homeostasis. *Biochem Biophys Res Commun.* 2012;422(3):393–7. 10.1016/j.bbrc.2012.04.160 22575514 PMC3376708

[45] Vink JY Charles-Horvath PC Kitajewski JK: Anthrax toxin receptor 2 promotes human uterine smooth muscle cell viability, migration and contractility. *Am J Obstet Gynecol.* 2014;210(2):154.e1–154.e8. 10.1016/j.ajog.2013.09.030 24060446 PMC3951982

[46] Yang MY Chaudhary A Seaman S: The cell surface structure of tumor endothelial marker 8 (TEM8) is regulated by the actin cytoskeleton. *Biochim Biophys Acta.* 2011;1813(1):39–49. 10.1016/j.bbamcr.2010.11.013 21129411 PMC3014418

[47] Gu J Faundez V Werner E: Endosomal recycling regulates anthrax toxin receptor 1/tumor endothelial marker 8-dependent cell spreading. *Exp Cell Res.* 2010;316(12):1946–57. 10.1016/j.yexcr.2010.03.026 20382142 PMC2886593

[48] Wajntal A Koiffmann CP Mendonça BB: GAPO syndrome (McKusick 23074)--a connective tissue disorder: Report on two affected sibs and on the pathologic findings in the older. *Am J Med Genet.* 1990;37(2):213–23. 10.1002/ajmg.1320370210 2248288

[49] St Croix B Rago C Velculescu V: Genes expressed in human tumor endothelium. *Science.* 2000;289(5482):1197–202. 10.1126/science.289.5482.1197 10947988

[50] Rogers MS Christensen KA Birsner AE: Mutant anthrax toxin B moiety (protective antigen) inhibits angiogenesis and tumor growth. *Cancer Res.* 2007;67(20):9980–5. 10.1158/0008-5472.CAN-07-0829 17942931

[51] Jinnin M Medici D Park L: Suppressed NFAT-dependent VEGFR1 expression and constitutive VEGFR2 signaling in infantile hemangioma. *Nat Med.* 2008;14(11):1236–46. 10.1038/nm.1877 18931684 PMC2593632

[52] Cao C Wang Z Huang L: Down-regulation of tumor endothelial marker 8 suppresses cell proliferation mediated by ERK1/2 activity. *Sci Rep.* 2016;6:23419. 10.1038/srep23419 26996335 PMC4800672

[53] Maurya SK Tewari M Kumar M: Expression pattern of tumor endothelial marker 8 protein in gallbladder carcinomas. *Asian Pac J Cancer Prev.* 2011;12(2):507–12. 21545221

[54] Gong Q Liu C Wang C: Effect of silencing TEM8 gene on proliferation, apoptosis, migration and invasion of XWLC‑05 lung cancer cells. *Mol Med Rep.* 2018;17(1):911–917. 10.3892/mmr.2017.7959 29115620 PMC5780170

[55] Opoku-Darko M Yuen C Gratton K: Tumor endothelial marker 8 overexpression in breast cancer cells enhances tumor growth and metastasis. *Cancer Invest.* 2011;29(10):676–82. 10.3109/07357907.2011.626474 22085271

[56] Gutwein LG Al-Quran SZ Fernando S: Tumor endothelial marker 8 expression in triple-negative breast cancer. *Anticancer Res.* 2011;31(10):3417–22. 21965755

[57] Høye AM Tolstrup SD Horton ER: Tumor endothelial marker 8 promotes cancer progression and metastasis. *Oncotarget.* 2018;9(53):30173–30188. 10.18632/oncotarget.25734 30046396 PMC6059023

[58] Greither T Wedler A Rot S: CMG2 Expression Is an Independent Prognostic Factor for Soft Tissue Sarcoma Patients. *Int J Mol Sci.* 2017;18(12): pii: E2648. 10.3390/ijms18122648 29215551 PMC5751250

[59] Ye L Sun PH Malik MF: Capillary morphogenesis gene 2 inhibits growth of breast cancer cells and is inversely correlated with the disease progression and prognosis. *J Cancer Res Clin Oncol.* 2014;140(6):957–67. 10.1007/s00432-014-1650-2 24667935 PMC11824005

[60] Ji C Yang L Yi W: Capillary morphogenesis gene 2 maintains gastric cancer stem-like cell phenotype by activating a Wnt/β-catenin pathway. *Oncogene.* 2018;37(29):3953–66. 10.1038/s41388-018-0226-z 29662192 PMC6053357

[61] Tan J Liu M Zhang JY: Capillary morphogenesis protein 2 is a novel prognostic biomarker and plays oncogenic roles in glioma. *J Pathol.* 2018;245(2):160–71. 10.1002/path.5062 29473166

[62] Cryan LM Rogers MS: Targeting the anthrax receptors, TEM-8 and CMG-2, for anti-angiogenic therapy. *Front Biosci (Landmark Ed).* 2011;16:1574–88. 10.2741/3806 21196249 PMC3066103

[63] Chaudhary A Hilton MB Seaman S: TEM8/ANTXR1 blockade inhibits pathological angiogenesis and potentiates tumoricidal responses against multiple cancer types. *Cancer Cell.* 2012;21(2):212–26. 10.1016/j.ccr.2012.01.004 22340594 PMC3289547

[64] Frankel AE Carter C Kuo SR: TEM8 targeted cancer therapy. *Anticancer Agents Med Chem.* 2011;11(10):983–92. 10.2174/187152011797927643 22023048

[65] Ye L Sun PH Sanders AJ: Therapeutic potential of capillary morphogenesis gene 2 extracellular vWA domain in tumour‑related angiogenesis. *Int J Oncol.* 2014;45(4):1565–73. 10.3892/ijo.2014.2533 24993339

[66] Bachran C Leppla S: Tumor Targeting and Drug Delivery by Anthrax Toxin. *Toxins (Basel).* 2016;8(7): pii: E197. 10.3390/toxins8070197 27376328 PMC4963830

[67] Szot C Saha S Zhang XM: Tumor stroma-targeted antibody-drug conjugate triggers localized anticancer drug release. *J Clin Invest.* 2018;128(7):2927–43. 10.1172/JCI120481 29863500 PMC6025988

[68] Byrd TT Fousek K Pignata A: TEM8/ANTXR1-Specific CAR T Cells as a Targeted Therapy for Triple-Negative Breast Cancer. *Cancer Res.* 2018;78(2):489–500. 10.1158/0008-5472.CAN-16-1911 29183891 PMC5771806

[69] Go MY Chow EM Mogridge J: The Cytoplasmic Domain of Anthrax Toxin Receptor 1 Affects Binding of the Protective Antigen. *Infect Immun.* 2009;77(1):52–9. 10.1128/IAI.01073-08 18936178 PMC2612287

[70] Wang SC Ye LIN Sanders AJ: Tumour endothelial marker-8 in wound healing and its impact on the proliferation and migration of keratinocytes. *Int J Mol Med.* 2016;37(2):293–8. 10.3892/ijmm.2015.2434 26677171 PMC4716791

[71] Castanon I Abrami L Holtzer L: Anthrax toxin receptor 2a controls mitotic spindle positioning. *Nat Cell Biol.* 2013;15(1):28–39. 10.1038/ncb2632 23201782

[72] Whitfield A Robinson AH: A Further Report on the Remarkable Series of Cases of Molluscum Fibrosum in Children communicated to the Society by Dr. John Murray in 1873. *J R Soc Med.* 1903;86:293–302. 20896998 PMC2037211

[73] Schussler E Linkner RV Levitt J: Protein-losing enteropathy and joint contractures caused by a novel homozygous ANTXR2 mutation. *Adv Genomics Genet.* 2018;8:17–21. 10.2147/AGG.S159077 30050362 PMC6057141

[74] Ahmed B Gritli S: Telogen hair loss and androgenetic-like alopecia in GAPO syndrome. *Australas J Dermatol.* 2019;60(2):e142–e144. 10.1111/ajd.12937 30255493

[75] Hu K Olsen BR Besschetnova TY: Cell autonomous ANTXR1-mediated regulation of extracellular matrix components in primary fibroblasts. *Matrix Biol.* 2017;62:105–14. 10.1016/j.matbio.2016.12.002 28011198 PMC5478475

[76] Liu S Crown D Miller-Randolph S: Capillary morphogenesis protein-2 is the major receptor mediating lethality of anthrax toxin *in vivo*. *Proc Natl Acad Sci U S A.* 2009;106(30):12424–9. 10.1073/pnas.0905409106 19617532 PMC2718377

